# In their absence; intensive care nurses’ experiences of communicating and supporting relatives from a distance

**DOI:** 10.1186/s12912-023-01559-4

**Published:** 2023-11-10

**Authors:** Helen Conte, Åsa Dorell, Emilia Wedin, Jeanette Eckerblad

**Affiliations:** 1 Department of Neurobiology, Care Sciences and Society, Karolinska Institutet, Alfred Nobels Allé 23, Huddinge, Stockholm, SE-141 83 Sweden; 2 Intesive Care Unit, Örnsköldsvik Hospital, Örnsköldsvik, 89189 Sweden

**Keywords:** Family, Intensive care units, Nursing, Pandemics, Qualitative research

## Abstract

**Background:**

Having a critically ill family member in the intensive care unit (ICU) is a challenging situation and ICU nurses play an important part in supporting relatives to make sense of the situation. Strict visiting policies inhibited the family’s presence in ICUs during 2020–22, and the communication between nurses and families changed drastically. Information and support are at the core of the ICU nurses’ profession, and the pandemic backdrop created a split between what intensive care nurses have a professional responsibility to perform and which actions were possible. To get a fuller picture, the aim of this study was: To describe intensive care nurses’ experiences of communicating and supporting relatives from a distance while working during visiting restrictions.

**Method:**

A qualitative descriptive design using individual and semi-structured interviews with 16 ICU nurses. The interviews were analyzed using inductive thematic analysis. This study followed the consolidated criteria for reporting qualitative research (COREQ).

**Results:**

Due to the visiting restriction during the COVID-19 pandemic, ICU nurses found themselves in a situation where proximity and time to develop an interpersonal connection with relatives disappeared overnight. The nurses’ experiences of communicating with and supporting families is described in three themes: “Finding ways to create order out of chaos”; “Guiding the relatives to a fuller picture of the situation”; and “Feeling insufficient in their support”.

**Conclusion:**

Visiting restrictions in the ICU meant that ICU nurses missed vital information about their patients as a person, which might have had a negative effect on personalizing and centring the patient care. But using a combination of digital and audio tools helped nurses to guide the relatives to a clearer picture of the situation as a whole. The support that nurses were able to provide to relatives was often insufficient due to the visiting restriction and as a consequence, they experienced physical and psychological stress.

**Supplementary Information:**

The online version contains supplementary material available at 10.1186/s12912-023-01559-4.

## Background

To have a family member in an intensive care unit (ICU) is associated with a risk for developing anxiety, depression, acute and post-traumatic stress disorder, especially if the patient’s diagnosis indicates a poor outcome [1–4]. Proximity to the critically ill person, clear, consistent, and understandable information are essential needs for family members to make sense of the situation [5, 6]. Healthcare staff in the ICU rate information and support as vital for family satisfaction in care [6]. The quality of how information and support is communicated and structured by healthcare staff is important for the family to maintain hope, trust, and to cope with the situation [5, 6]. The healthcare staff´s supportive communicative strategies consist of individualizing information, preparing the family, and supporting them in the process of understanding the situation and their loved one’s health status. Non-supportive behaviours consist of avoiding, distancing, and hindering families’ access [7].

Providing information and support are at the core of forming interpersonal relationships in which ICU nurses play an important role by identifying the relatives’ needs and by integrating these in person-centred care [5–7]. The pandemic backdrop, 2020–2022, created a split between what intensive care nurses have a professional responsibility to perform, and which of those actions it was possible to maintain. Research from the initial phase of the COVID-19 pandemic described a situation where nurses stumbled into chaos, where quality of care was diminished [8]. Nurses in the ICU were faced with an increased workload, an influx of critically ill patients, and the separation and isolation of patients from their family members [8–10]. Researchers debated from the beginning of the pandemic that families had to remain a critical focus in the ICU, and for nurses to maintain their support of family members [11–13]. The visitation restrictions affected the family’s presence in the ICU [8–10], and sometimes it included end-of-life care [10, 14].

Gradual and local adaptation to visitation policies in the ICUs occurred to various extents in Sweden, Finland, and Norway during 2020–21, with research suggesting that professional standards of family care were compromised [8, 9]. Nurses adapted to the situation during the initial part of the COVID-19 pandemic [8–10]. One study suggested that nurses became a proxy for the relatives, but the meaning of the concept was not explored further, nor how the nurses adapted and found ways to support relatives [8].

Research synthesized from respiratory pandemics has suggested that when restrictions and isolation were imposed, communication between healthcare staff and families changed drastically [15]. Nurses were ambiguous about families being present in the ward due to the risk or fear of spreading the infection, and of protecting patients, families, and themselves [8–10, 15, 16]. The interpersonal communication changed who communicated with the family, what measures were used, what was communicated, and how often [15]. Visiting restrictions led to diverse technological and team-based solutions to uphold contact with relatives [15, 17–19].

Research has begun to describe problems, variations, and limitations concerning how information and support was communicated and structured. To adapt actions in practice entails learning from experiences and contextual conditions. More knowledge is needed concerning the adaptation of strategies that the nurses practiced during the different waves of the pandemic to support families whose presence in the ICU and proximity to the patient was limited. The aim of this study was therefor to describe intensive care nurses’ experiences of communicating and supporting relatives from a distance while working during visiting restrictions.

## Method

A qualitative descriptive design using individual and semi-structured interviews were chosen to capture the nuanced experiences of the participants. The interviews were analysed with thematic analysis [20, 21]. This study was conducted and reported in adherence to the consolidated criteria for reporting qualitative research guidelines (COREQ) [22]. The result of this study has not been published before.

### Study setting

Four Swedish intensive care departments were included in study. The departments were located in different geographical regions, two university hospitals, one large inner-city hospital, and one small inner-city hospital.

### Participants and recruitment

The participates were included through a convenience sample. The inclusion criteria were nurses with a postgraduate education in intensive care, working in ICUs during the COVID-19 pandemic.

The heads of the four ICU departments were approached with written information about the study and gave their written consent to conduct the study in their departments. Prospective participants received verbal and written information regarding the study from the head nurses during staff meetings. ICU nurses who were interested to participate and met the inclusion criteria, registered for the study by signing an informed written consent. The Swedish Ethical Review Authority in Gothenburg approved the study (DNR 2020–05961). Nineteen ICU nurses were contacted by email by the research group. Participants were given the opportunity to choose the date and time of day for the interviews.

### Data collection

A semi-structured interview guide was constructed for this study. The authors tested the interview guide during the first interview and concluded that no change was necessary. The guide had two main areas that were represented with questions. The first was “Would you like to describe how communication with relatives worked during the COVID-19 pandemic?” And the second was “What strategies did you use to communicate, support and inform relatives during the COVID-19 pandemic?” The authors used follow-up questions, such as “How do you think it worked?”, “Do you want to describe it?”, and “How did that feel?” The questions involved in the interviews are detailed in Additional file 1. The interviews were conducted by all authors (HC, ÅD, EW, JE) through video conferences between May and December 2021. Video conference as a method to preform interviews were chosen due to visitor restriction of all hospitals at the time and the wide geographical diversity of the included hospitals. To perform qualitative interviews thru video conference have shown to be a good complement and comparable to face to face interviews [23]. All authors are ICU nurses with previous clinical experience; three (HC, ÅD, JE) are university lecturers, and one (EW) a clinical nurse in ICU. All authors have previous experience of conducting qualitative studies and were involved in conducting the interviews, but with no prior contact with the respondents. The initial question in the guide focused on getting a description of the demographic data of the participant, the ICU and the situation they worked in during 2020–2021. The length of the interviews varied between 20 and 56 min (median 41 min), were audio recorded, and transcribed verbatim. The transcription was coded before being shared with the entire research team. Sufficient information power was reached after 16 interviews. Information power are based upon the specificity of the aim and sample, quality of the dialogue, and strategy of the analyses [24].

### Data analysis

The interviews were analyzed using inductive thematic analysis, with focus on identifying patterns and meaning within a rich data set [20, 21]. The process consists of six steps. In the first step, each author re-read their interviews several times, taking notes and marking initial passages to familiarize themselves with the data. In the second step, each author coded the interviews within the same dataset and identified data relevant to the codes. A group discussion was held in the third step to describe recurring patterns between each group of four. In the fourth step, two authors (JE and ÅD) read the transcripts again and developed a structured analysis framework with primary codes, which were reviewed in the research group. Coded extracts from the entire data set were independently generated by the two and grouped into potential themes. The co-authors refined the themes together. In the fifth step, one author (JE) continued to name and defined the themes. The final structure was created after all authors had checked and reviewed the data. Two authors (JE and HC) reviewed and condensed the themes to three, with six sub-themes. The sixth and final step was to produce the report.

## Result

The result is based on interviews with 16 ICU nurses, 12 women and four men. Three nurses withdrew their participation. The participants had a median of nine years’ work experience, with a range of 1–28 years as a nurse in the ICU. During the analysis of the interviews, three main themes emerged: “Finding ways to create order out of chaos*”;* “Guiding the relatives to a fuller picture of the situation*”; and “*Feeling insufficient in their support*”.*

### Finding ways to create order out of chaos

The theme describes how nurses went from being overwhelmed by the large influx of critically ill patients in the ICU´s and how conditions changed the ways nurses upheld contact with the relatives. The theme includes two sub-themes: “*To find ways to be in contact with the relatives*” and “*To adhere to but increasingly question visiting restrictions*”.

#### To find ways to be in contact with the relatives

The participants described that the situation in the ICU was initially chaotic, the phones were constantly ringing, and no one had time to answer. The participants understood how terrified the relatives were, and how eager they were to get information of how their family member were doing. The ICU nurses in charge of the patient were bedside, isolated in full protective suits, and unable to take the phone calls, due to either the severity of the patient’s health condition, lack of protective equipment, or both. It was impossible for them to hear and talk on the phone while wearing this equipment.

*“And when you got a call, it was almost impossible to talk or hear through the protective equipment.” (Participant 6)*

Routines concerning when the relatives should or could be contacted, and who should contact them, were not in place from the beginning. The lack of clear routine led to relatives sometimes having to wait several hours before the nurse in charge of their family member was replaced by staff from the next shift and able to make a phone call. The participants understood that this was a long time to wait for information, and that the relatives were stressed about not knowing how their family members were doing. To make things work, new routines were incorporated into the organizations regarding who should initiate and uphold the contact with the relatives. The routines entailed who, how and how often this contact should take place. The ICU nurses described that they had to let this part of their work go in favour of caring for the patients.

*“It felt like the whole community was thrown down to the deep end of the pool and someone said, “now you need to swim”. So, I think, we had to learn or find out how to support relatives and what way to pedagogically convey this transition from someone being healthy to someone being critically ill in the intensive care.” (Participant 16)*

Relatives were often offered contact with a counsellor for support. The anaesthesiologist in charge or a nurse functioning as coordinator contacted the families with updates on a daily basis, either by phone (audio only) or video calls.

*“And then the anaesthesiologists suggested that they could call the relatives to inform them once a day. That was much appreciated by the relatives, and you noticed a difference directly. When the doctors called once a day our phones stopped ringing and we could get back to focusing on the patients without being disturbed by the phone all the time.” (Participant 12)*

Because of this routine the nurse in charge of the patient no longer had the responsibility to keep contact with relatives on daily bases, they initially felt relieved since it decreased their workload to some extent.

*“I remember that, in the beginning I was relieved that the relatives were not here to see this. I was relived because, I would never have had the time to explain anything or to support them.” (Participant 8)*

However, the ICU nurses increasingly found themself lacking important information about their patients. They missed finding out the things which the relatives often shared when they were present and bedside in the ICU regarding the patient as a person. The nurses expressed they missed information which they could use to connect with and guide the patient back to reality when they woke up in an unknown environment among unknown people.

*“Well, yes, it was like a little bit, or no, a big piece was lost. Because, what you know about your patient, what you have learned about the person, what interests and other things like that, things I usually find out when relatives are visiting. If you have worked with patients when they are waking up, you can use that information in order to guide them back to reality. But it got lost, it disappeared there and then.” (Participant 6)*

#### To adhere to but increasingly question visiting restrictions

In the beginning of the pandemic, the COVID-19 disease was still unknown, and the ICU nurses had both limited knowledge about the disease and experiences in caring for patients with the disease. During the first wave, the ICU nurses expressed having no doubts about the importance of maintaining the visiting restrictions. These were necessary due to lack of personal protective equipment. The ICU nurses narrated that even working in full protective suits, they had to be very careful to limit the risk of being infected themselves and to avoid spreading infection to others.

The influx of critically ill patients admitted to the ICU was so extensive that it caused a lack of beds, space, and staff, which the participants suggested hindered family members from being present in the ICU. Due to the high workload, participants described that they did not have time to deal with relatives, even if they had been allowed to visit. Due to reports from the media regarding the chaotic situation in the ICUs, the relatives accepted the visiting restrictions and that it sometimes took a long time to get information about their family member. They were grateful when someone from the medical staff had the opportunity to call them.

*“WelI. yes... but since there were so many articles about ICU in the media, all the relatives were grateful that we even had time to call them. So, in that way, it kind of made it easier for us, I have to say.”* (Participant 15)

Some of the participants began to have mixed feelings about the visiting restrictions after the first wave, when things started to settle down a bit and there was no longer a lack of protective equipment. They felt sorry for keeping the families apart but at the same time they did not feel they had the time or strength to have them back on the ward. In some cases, the patient could be in ICU for several weeks and up to a couple of months, and they understood the trauma of families being separated from each other.

*“Well, normally they come to visit for shorter or longer periods of time, but now that it has been like it is, sometimes you can feel that it is a good thing not to have relatives around, I would not have time for them. At the same time, it’s hard for the patients not to get any visits.” (Participant 3)*

As time passed, more nurses started to question the strict no-visit policy. During the third wave, some of the relatives who called the ICU and knew that they have had COVID-19, had antibodies, or were vaccinated, were really upset by the restrictions. The participants described that there were situations when the relatives simply did not accept the no-visit policy anymore, and they had to argue with relatives who were very upset. On the one hand, they knew why they had to say no, but on the other hand, they could hear how devastated the relatives were.

*‟The hard part is to hear how desperately they want to come here, and it puts pressure on me, – ‘please, can’t we just, for a little while? and especially now – it is starting to be … I’m vaccinated, and I have antibodies...’ So, of course ... But still, you have to refer to the policy.” (Participant 2)*

At that point, some of the ICU nurses started to actively question the rigidity of the restrictions. They did not believe the visiting restrictions were adapted to the current situation since there was no longer a lack of protective equipment. The participants described that it was harder to maintain the same argument about why the relatives could not visit, especially listening to the relatives’ stories and understanding how much they suffered, how sad, worried, and upset they were.

*‟And then it became much more difficult, the relative could say, ‛I’m fully vaccinated and you in the staff are as big a risk to him as I am.” (Participant 2)*

Another consequence of the no visiting restrictions was that the participants felt they missed opportunities to get to know their patients as a person through the interactions with the family, such as who they were before they became severely ill.

### Guiding the relatives to a fuller picture of the situation

The theme describes how nurses used all means possible to support the relatives to make sense of the situation and being a part of the patients´ care. This theme emerged out of two sub-themes,* “To be the eyes of the relatives*” and “*To find technical solutions”.*

#### To be the eyes of the relatives

Phone conversations with the relatives were often short and the main focused were information on the patients’ current health condition. The participants described that an unproportional large part of the conversation focused on vital signs, such as temperature, pulse, blood pressure, respiratory rate and oxygen saturation. This focus often originated from questions from the relative, such as – “how is the fever today?” The ICU nurses understood that fever was something that the relatives were familiar with. For a person with no previous experience of intensive care, to understand what the patient went thru, the patients’ health status and changes in their condition would be difficult to grasp. So, in order to give the relatives a fuller picture some of the participants described how they had developed a strategy and tried to help the relatives visualize how the patient and the surroundings, e.g., the room, the high-tech equipment, monitors, looked.

*“I kind of start at one end, by describing what things looks like and end at the other. Not just what the patient looks like but the picture I image that they don’t have. Things that could be important in order to understand.” (Participant 1)”*

The participants thought it was challenging to explain and guide the relatives to understand severity of the patients’ health condition over the phone since they relatives could not use visual or auditory senses to understand. The ICU nurses spent time and used a whole range of cues to support the relatives to visualise the patient´s current situation and health condition. By visualizing and describing what they saw they could be the eye of the family members.

*“Because it’s hard to convey how serious the situation is when you can’t show people.” (Participant 16)*

*“So it’s probably more that you need to be the eyes of the relatives, to describe what things look like around the patient and also what the patient, who could be their husband, father or mother looks like now. You must explain everything, tape and hoses and cords and, well, it’s more so, that I’m their eyes inside the patient’s room.” (Participant 2)*

#### To find technical solutions

Most calls between the relatives and the healthcare providers were made by audio calls; video calls were sparse and not something that the participants expressed they pursued on a regular basis. Not being able to meet or see each other created an obstacle in the communication between the ICU nurses and the relatives. Being unable to see the person they spoke to, made the ICU nurses unsure, since it was hard not knowing how the person on the other end of the phone reacted to the information they gave.

*‟No but, it won’t be the same when it’s just on the phone. Now, I don’t know what the relative looks like. The look can express different things. You can look at a relative and see if they look concerned, or express fear or if they don’t understand anything, but through phones it becomes much more difficult.” (Participant 1)*

When the participants talked to the relatives before the patients left the ICU, they experienced that the relatives sometimes had a hard time understanding how severe the patient’s condition had been, and what they been through; something that they thought might affect the recovery for them both. Before the pandemic, a patient this ill would normally have had a diary, which the participants pointed out would have been a good thing for both the patient and the relatives at this point to illustrate and explain the situation. However, because of the high workload during this time, diaries for patients were not often prioritized. As a result of the visiting restrictions, video calls and/or text messages were introduced to various extents in all four ICUs. The technique and equipment differed between the units and there was a diversity in how often video calls with relatives were used, and who participated in these calls.

*“We most often used regular phone calls, sometimes Facetime and in those cases where the patient was awake and able to make a decision if he/she wanted to join.” (Participant 1)*

The participants from one ICU used the term ‘video visits’ and had developed a structured approach to guide and support the relatives and the patients. One person in the unit took responsibility for the overall planning, with setting times, starting the calls, guiding the families in the ICU environment, and introducing them to the bedside ICU team. These video visits were a good alternative for the relatives to meet/see the healthcare provider or the patient, and to get some information at the same time, even if they lived in different parts of the country. It also gave the healthcare provider a chance to show and explain the close environment, the equipment, and put this in relation to the situation in which the patient was cared for.

*“We started to use video calls eventually, I can´t say exactly when it started but I know that we had a whole family connected on video calls, via iPad or tablet. It was good to have the relatives more involved in the process and to see the patient.” (Participant 9)*

Participants at other ICUs described having discussions among the staff about video calls between relatives and patients, and they had come to a mutual agreement that the patient should be awake and able to agree upon taking and participating in the call. They did not want to show the patient in a sedated state.

A regular audio call between the patient and the relative was also used, even if the patient could not talk but was awake. In those cases, the patient would get help from one of the healthcare providers, holding the phone to the ear and helping to convey what was communicated between the relative and the patient. This was one way for the relative and the patient to get some contact, and for the patient to hear the voice of their family members. To connect a video call took planning and organizing before the call could be set up, and often the participant nurses did not have time to do it. Some ICU departments used video calls and text messages more frequently; they had one person, a coordinator in charge of organizing and scheduling the video calls for the relatives to all patients.

*‟But that I wish it could be done more easily. That we could have video calls in a smoother way instead of having to learn how to schedule meetings in calendars and send out links and ... Yes, it was not a success because it was too cumbersome. And it was all on me, but, if we had got help to set it up, then …” (Participant 13)*

Digital calls or facetime were, however, not often used when it came to the daily calls between the healthcare provider and the relatives. For those, the regular audio phone was used.

### Feeling insufficient in their support

The theme describes how nurses struggled with feelings of guilt and powerlessness since they were not able to offer the care and attention the patient and their relative needed and deserved. The theme contains two sub-themes: *“to lack the interpersonal connection”* and* “to experience physical and psychological stress”.*

#### To lack the interpersonal connection

The participants described difficulties in creating a connection with the relatives when they communicated on the phone. To build a nurse-relative interpersonal connection was considered a process that takes time, and this phenomenon disappeared overnight due to the pandemic. Before the restrictions, relatives were often bedside, which gave an opportunity to build a relationship and to create hope and trust between the relatives and the nurses. During the restriction, the relatives were often reduced to just a name, which sometimes led to distancing.

*‟What you miss is all that small talk with the relative beside the patient’s bed, where you will find out more about what the patient likes, they will tell you anecdotes from their life. You rarely have that time now, when you’re just going to make that phone call somewhere during the day when you have a minute, it has often become very clinical.” (Participant 2)*

The nurses experienced that it was harder to meet the relative’s needs, to inform and to get a sense of their emotional status based on their verbal reactions.

*“I can see their body language and I see their facial mimicry when they come into the room, and I can catch them.” (Participant 15)*

Some situations were more difficult than others; the participants described situations when a withdrawal of treatment was decided, and the relatives were allowed a last visit to say farewell. To meet the relatives in person for the first time when there was no hope of recovery and it was no longer possible to maintain life, was difficult. The participants described it as more difficult to give emotional support and consolation to a relative they had only talked to on the phone. This rarely occurred before the pandemic, but now it was more frequent, and it was hard to witness time after time.

*“It’s not the same way; normally if you have the patient who has been here for a long time, you might have met the relatives five, 10, 15 times before you have that talk, that there’s no hope of recovery, we need to end the treatment. And now, it may be the first time you meet them, when they are going to get that message.” (Participants 2)*

#### To experience physical and psychological stress

The participants knew what was expected of them and what they expected of themselves, but they did not have the ability or the strength to do what they knew was right. Some of the participants described that sometimes, even if they had time to return that call at the end of their working day, they just could not do it; they simply did not have any strength left. They were too exhausted to call, and this added to an already troubled conscience.

*‟Well, I don’t think I’ve ever seen people (my co-workers) as tired as they are right now, you just can’t take those extra steps to make that call to the relative at the end of your day.” (Participant 1)*

The participants experienced stress knowing that the relative was sitting at home waiting for their call. Confronted with these stressful thoughts, they often felt powerless and that they were not fulfilling their obligations as a nurse. Not being able to call back, not giving the support and consolation they knew the relative needed, added to the stress the nurses were already experiencing.

*“I probably tried not to get involved emotionally, just to get by and try to cope. Because it was extremely hard.” (Participant 14)*

The feelings could be so difficult to manage that some of the nurses tried to turn their emotions off or block them out. Other nurses described that they had memory gaps from the first part of the pandemic.

*“I’ve learned to repress my emotions, when I start talking about it, I realize that I actually remember a lot that I didn’t think I did, but I have a lot of memory gaps during the first year. Extreme memory gaps with only a few glimpses. But when we talk about it, it starts to come back.” (Participant 9)*

## Discussion

The participants in the current study described the situation in 2020 as like stumbling into a virtual and emotional chaos where the amount of critically ill patients, lack of beds and staff led to limited possibilities for ICU nurses to support relatives of the critically ill patients. To encourage physical proximity was not initially a possibility that the participants considered, and time to offer structured information and support over the phone was limited. The 16 participants in the current study worked in diverse geographical regions and were faced with a whole range of personal, interpersonal, and contextual factors that led to the necessary but traumatic separation and isolation of patients from their relatives, which is confirmed by ICU nurses in other studies [8–10, 14, 25]. The extent and experiences of the strict non-visit policies varied on a national and regional level [8, 9]. Like the four ICUs in the current study, other ICUs in Sweden, Norway and Denmark had limitations; 100% of all Swedish ICUs had non-visit policies in parts of 2020, and the adaption to restricted visiting varied [9].

The participants described thinking about, understanding, but also sometimes shutting out, the thoughts of the relatives’ fear, trauma, and suffering. This was initially a way of being able to handle the patients and situations in the ICU. After moving on from the initial chaos, the ICU nurses described different ways to create order, finding ways to establish contact with the relatives and a range of actions where they tried to guide the relatives to get a fuller picture of the patient, the situation, and the ICU environment. They experienced that without proximity and time to develop an interpersonal connection with the relatives, they missed vital information about the patient as a person. The relatives were sometimes reduced to being a name and not the one by the nurses’ side guiding the patient back to reality in a strange and unreal environment. Participants in the current study, as well as nurses in other studies, have described the changing support and information that the relatives received as a process of adaptation; their own work was negotiated and adapted to factors due to the current situation [8, 10, 15, 16].

The participants described understanding how terrified the family members were; believing it was unacceptable for relatives having to wait for information, and their own workloads, were some of many factors which led to new routines concerning information and support being incorporated into the four different ICUs. The anaesthesiologist or the nurse functioning as a coordinator took over and made short daily phone calls to the family. Counsellors had regular contact with relatives to coordinate emotional and social support. The nurses closest to the patients felt that they missed valuable information about the patient as a person, and they were not always in the loop on what was being said, how and when information was communicated. Drawing on research pre-dating COVID-19 and from other respiratory pandemics, the relatives suggest it is vital how, when and who provides information and support [5–7, 26]. Synthesized research suggests that who upheld contact, structure and tools for communication differed, but a common theme was that communication focused mainly on the patient’s physical condition and short-term progress, and that the relative’s involvement was limited [15].

The nurses in the current study felt that their support was insufficient, some of them experienced physical and psychological stress since they knew that relatives were waiting for somebody to call. Several of the participants believed that as specialist nurses they failed the relatives by not having the time to create an interpersonal connection, which is the foundation support is built on. Nurses, both in the current study and others, were overwhelmed initially but adapted to not having relatives in the hospital [8, 10], but they experienced dilemmas of depersonalization [15], and that standard of good care was affected [8, 15]. The participants in the current study described that it became increasingly difficult to adhere to the visiting restrictions with time. They experienced that the rigid restrictions, not always adapted to the changing situation, and depriving relatives and patients of proximity, perpetuated their trauma and suffering. These experiences are validated by relatives and nurses in other research, where proximity, information, and support have been identified as essential needs and vital for helping relatives to cope and make sense of the situation [5–7, 25]. Healthcare staff also suggest that assurance is an essential need for the family, and interventions such as written information and family support meetings can play an important part in coping and sensemaking [5, 6].

A person critically ill with COVID-19 in the ICU destabilizes the entire family [14], and relatives suggest clear and consistent information is vital to make the situation predictable and to enable them cope [5, 6, 15, 18, 25]. The distinction between when information ends and support begins can be, at times, muddled since the concepts are interconnected but relatives suggest that the support of the healthcare staffs is crucial for them to maintain trust and hope [5, 6, 15, 18, 25]. Nurses can serve as important information and communication facilitators [5, 6, 15]. What and how something is being communicated contributes to the relatives’ sensemaking. The participants in the current study began to regain their work of supporting and guiding the relatives to get a fuller picture of the situations through audio or video calls. A fuller picture included communicating information and describing the situation, i.e., dimensions of the patient’s condition, the environment, and the teams’ work. They were trying to compensate for the relatives not being able to see, hear, smell and feel the patient and took time to guide the relatives by describing and framing the patient’s situation in the ICU. The challenges they experienced of not being able to see the relatives, their reactions and emotional response, is confirmed as a central theme in research [14, 17, 18, 25]. Trying to compensate for the relatives lost ability to use their senses in grasping the situation, many of the participants described how they took time and guided the relatives from known concepts like fever and respiratory rate, to beginning to sense a fuller understanding about the severity of the patient’s health condition. From other studies it is known that relatives describe being absent from the ICU as not being able to observe, feel and participate. They needed to seek and confirm the situation by searching for more information [14]. The information was, at times, inconsistent, arbitrary and in a language, they did not understand [5, 6, 14, 18, 26].

The families’ need for assurance in their sensemaking indicates that the quality of the interpersonal communication with the healthcare staff is a vital factor [5, 6]. A supportive nurse-family relationship can foster a positive experience and reduce negative experiences when the family goes through phases of shock, disorientation, turmoil and altered family dynamics [26]. The nurses’ experiences in the current study suggests that they used a range of actions, from supportive to non-supportive behaviour. They, as confirmed by other research, ranged from using strategies to make the situation understandable and facilitating communication, to avoiding the issue and limiting participation [18].

The current study suggests regional, local, and personal variations in finding and using communicative technical solutions to communicate information and structure support. Audio calls were commonly used, and they used a range of systems for making video calls. The experiences of nurses in one ICU differed from the rest; video calls were used in combination with audio calls and text messages. The nurses from that ICU used the term ‘video visits’ and one person oversaw booking and planning the visits. That person also started up the video visits, guided the relatives through the environment, to the patient, and introduced the team present at the bedside. A range of solutions were used in other studies to provide support and information, including audio calls, video calls, family support teams [8, 14, 15, 17, 18, 27, 28]. Combinations of video and audio calls were used but audio calls were more common [17]; the interactions during video calls took longer and took more preparation, but were doable; however, as the current study demonstrates, a facilitator would have been beneficial [17, 28].

Looking at the relatives’ experiences from research, they suggest future technical solutions should mimic bedside communication [17], giving them a sense of being held [18]. The support and information needed to be regular, structured and predictable [14, 15, 25] and give them a chance to reach somebody with their questions [18]. During 2021–2022, regional and local limitations to the open visitation policies were still in place to reduce the spread of infection. This might be a permanent way of the future, where family support must be continually adapted, and it is therefore vital to apply individual and person-centred solutions based a combination of digital and audio tools to guide relatives to a clearer picture on what we know so far.

### Strengths and limitations

Trustworthiness of a qualitative study are related to the strength of the method, the credibility and transferability [29]. To strengthen the credibility, three of the authors (JE, HC, ÅD) discussed each step of the analysis process and reached a consensus regarding the subthemes and the themes and the process are described in the method section. To further strengthen credibility of the study several quotations are presented in the result. Participants included in this study worked in different parts of Sweden with hospitals and ICU wards of different sizes which strengthen the transferability.

A limitation in this study could be that the data collection had to be conducted online thru video conference, however during this period most meeting and conferences were done thru video conference and people got use to communication thru digital platforms. The data that resolved from the interviews were rich and with depth. The study was carried out midst the pandemic and the ICU nurses were overworked and fatigued. If the data collection had been performed later, when the pandemic had slowed down, there might have been more nurses signing up for the study. However, these ICU nurses’ willingness to share their experiences provided sufficiently rich descriptions, and even though only 16 nurses were interviewed, information power was well achieved after these interviews were performed. Another limitation of the transferability could be that this type of sampling could be biased in its selection process. The nurses who signed up for this study, despite of their heavy workload, might had a special interest in caring for the relatives.

## Conclusions

Visiting restrictions in the ICU during the COVID-19 pandemic led to nurses finding themselves in a situation where proximity and time to develop an interpersonal connection with relatives disappeared overnight. ICU nurses missed vital information about their patients as a person which they expressed might had a negative effect on personalizing and centring the patient care. The nurses experienced that their support were insufficient and that the relatives suffered due to the visiting restriction, isolated and separated from the critically ill patient in the ICU. Nurses started finding ways to communicate and support the families, where a combination of digital and audio tools helped them to guide the relatives to a clearer picture of patient health condition and the situation as a whole.

### Supplementary Information

**Additional file 1.** Interview questions.

## Data Availability

Data can be shared upon reasonable request. Request of data (pseudo-anonymized) can be sent to the Research Data Office (rdo@ki.se) at Karolinska Institutet, which will require a data transfer agreement and will be handled according to the relevant legislation in Sweden.

## Abbreviation

ICU: Intensive Care Unit
COREQ: Consolidated criteria for reporting qualitative research
