# Supplementary material for: In their absence; intensive care nurses’ experiences of communicating and supporting relatives from a distance
Source: BMC Nurs. 2023 Nov 10;22:421. doi: 10.1186/s12912-023-01559-4 (PMC10636861; doi:10.1186/s12912-023-01559-4)
Supplement: Supplementary file 1 — Additional file 1. Interview questions. [file 12912_2023_1559_MOESM1_ESM.pdf]

Additional file 1

Interview questions

Would you like to describe how communication with relatives worked during the COVID-19 pandemic?

follow-up questions

-How do you think it worked?

-What was your role?

- Were there other people involved?

-How did it feel?

-Can you describe how it worked?

-Do you remember any specific situation?

-What could have facilitated the communication?

-What were the challenges?

-How have this changed over the past years?

What strategies did you use to communicate, support and inform the relatives about your patients during the Covid-19 pandemic?

follow-up questions

-Can you describe it?

-How did it work?

-Where or how did you learn to do that?

-Who did you work with?

-Could you describe such a situation?

-How did it feel?

-Was there anything that could have facilitated your work?

-What hindered your work?
